# Multiscale Representation of Observation Error Statistics in Data Assimilation

**DOI:** 10.3390/s20051460

**Published:** 2020-03-06

**Authors:** Vincent Chabot, Maëlle Nodet, Arthur Vidard

**Affiliations:** 1 Univ. Grenoble Alpes, Inria, CNRS, Grenoble INP, LJK, 38000 Grenoble, France; vince.citron@gmail.com (V.C.); maelle.nodet@uvsq.fr (M.N.); 2 Université Paris-Saclay, UVSQ, CNRS, Laboratoire de Mathématiques de Versailles, 78000 Versailles, France

**Keywords:** data assimilation, observation errors, error correlation, multiscale analysis, wavelets, error covariance matrices

## Abstract

Accounting for realistic observation errors is a known bottleneck in data assimilation, because dealing with error correlations is complex. Following a previous study on this subject, we propose to use multiscale modelling, more precisely wavelet transform, to address this question. This study aims to investigate the problem further by addressing two issues arising in real-life data assimilation: how to deal with partially missing data (e.g., concealed by an obstacle between the sensor and the observed system), and how to solve convergence issues associated with complex observation error covariance matrices? Two adjustments relying on wavelets modelling are proposed to deal with those, and offer significant improvements. The first one consists of adjusting the variance coefficients in the frequency domain to account for masked information. The second one consists of a gradual assimilation of frequencies. Both of these fully rely on the multiscale properties associated with wavelet covariance modelling. Numerical results on twin experiments show that multiscale modelling is a promising tool to account for correlations in observation errors in realistic applications.

## 1. Introduction

Numerical weather prediction requires the determination of the initial state of the system in order to produce forecasts. Retrieving an optimal initial condition requires the use of so-called data assimilation methods that combine information from observations, model equations and their respective error statistics.

Since the late 1970s satellites have been a dominant source of information. Errors associated with such data are highly correlated in space and between different frequency channels, which can be detrimental if they are not accounted for, even approximately [1,2]. Due to the size of the observation vectors, building and handling corresponding error covariance matrices is not feasible in practice. Consequently most data assimilation systems assume that observations are uncorrelated with each other. This either induces severe misspecification of error statistics, or necessitates the use of only a fraction of the available observations to ensure this assumption to be valid [3]. Considering the high cost of remote sensing observation, this situation should be avoided. For this reason the representation of correlated observation errors has very recently become a significant topic of research and several routes are being explored. First research was directed to accounting of inter-channel error correlation. Due to the modest size of the resulting covariance matrix, the main problems lie in the poor quality of the error estimations and on the detrimental effect it has on the conditioning of the minimisation problem (see [4,5] and bibliography). For spatial correlation, a practical approach is to use a covariance matrix that is block diagonal. This is manageable when observations can be grouped into small enough batches which are uncorrelated with each other [6]. For a more general spatial distribution [7] proposes to represent convolutions with the covariance matrix by a diffusion equation discretized using a finite element approach.

On the other hand, when observation are dense in space, some (multiscale) transformations can be applied to the data in order to perform efficient subsampling of the observations [3]. Such transformations can also be used to permit a cheap but good approximation of said error statistics representation [2]. The latter approach, which is the main topic of this paper suffers from two main difficulties. First, dealing with partially missing data in one set of observation is not straightforward and requires a special treatment of observation error statistics in the frequency domain. Second, as mentioned above, considering spatially correlated observation errors can severely damage the convergence properties of the assimilation methods. In this paper, after a short introduction to the context of general data assimilation (Section 2.1) and wavelet representation of the observation errors (Section 2.2), we present its actual implementation in the main data assimilation techniques (Section 2.3 and Section 2.4) and discuss in detail the aforementioned difficulties Section 2.5. Proposed solutions are then implemented on a simple case mimicking a laboratory experiment (presented in Section 2.6), and their performance is discussed in Section 3.

## 2. Materials and Methods

### 2.1. General Formulation of Data Assimilation

Let M be a model describing of the dynamics in a given system, represented by its state vector x. For example, x might be a vector of temperatures over a grid (discretized area of interest).

(1) $$\frac{\partial x}{\partial t}{\left(t\right)=M\left(x\left(t\right)\right),\;x|}_{t=0}=x_{0}$$

where $x_{0}$ is the initial value of the state vector.

Data assimilation aims at providing an analysis $x^{a}$ which will be used to compute optimal forecasts of the system’s evolution.

Such an analysis is produced using various sources of information about the system: observations (measurements), previous forecasts, past or a priori information, statistics on the data and/or model errors, and so on.

In this paper, we assume that these ingredients are available:

- the numerical model M,
- a priori information about $x_{0}$, denoted $x_{0}^{b}$ and called *background state vector*,
- partial and imperfect observations of the system, denoted y and called *observation vector*,
- the observation operator H, mapping the state space into the observation space,
- statistical modelling of the background and observation errors (assumed unbiased), by means of their covariance matrices B and R.

Data assimilation provides the theoretical framework to produce an optimal (under some restrictive hypotheses) analysis $x^{a}$ using all the aforementioned ingredients. In this work, we will focus on how to make the most of the observation error statistics information and we will not consider the background error information. Regarding the observation information, typically, most approaches can be formulated as providing the best (in some sense) vector in order to minimize the following quantity, measuring the misfit to the available information:

(2) $${∥H\left(x\right)-y∥}_{R}^{2}$$

where the notation ${∥z∥}_{K}^{2}$ stands for the Mahalanobis distance; namely ${∥z∥}_{K}^{2}=z^{T}K^{-1}z$. Some information about algorithms and methods will be given in following paragraphs. For an extensive description we refer the reader to the recent book [8].

### 2.2. Spatial Error Covariance Modelling Using Wavelets

Being able to accurately describe the covariances matrices B and R is a crucial issue in data assimilation, as they count as main ingredients in the numerical computation. The B matrix modelling has been largely investigated (see e.g., [9,10]). DA works actually using non diagonal R matrices are quite recent (e.g., [2,7,11]). Evidence shows that observation errors are indeed correlated [12] and that ignoring it can be detrimental [13,14].

In [2] the authors introduced a linear change of variable A for accounting for correlated observation errors, while still using a diagonal matrix in the algorithm core. For the sake of clarity we will summarize the approach in the next few lines. If we assume that the observation error ϵ is such that $\epsilon =y-y^{t}$, with ϵ∼N(0,R), $y^{t}$ being the true vector (without any error) and N(0,R) designing the normal distribution of zero mean and covariance matrix R. Then changing variables writes $\beta =A\epsilon =Ay-Ay^{t}$ and $\beta ∼N(0,ARA^{T})$. Then we carefully choose A so that the transformed matrix is almost diagonal: $D_{A}=\mathrm{diag}\left(ARA^{T}\right)≃ARA^{T}$. Indeed, we then have the following property:

$$\begin{array}{c} {∥y-H\left(x\right)∥}_{R}^{2}={\left(y-H\left(x\right)\right)}^{T}R^{-1}\left(y-H\left(x\right)\right)\\ \;≃{\left(y-H\left(x\right)\right)}^{T}A^{T}D_{A}^{-1}A\left(y-H\left(x\right)\right)={∥Ay-AH\left(x\right)∥}_{D_{A}}^{2} \end{array}$$

After this change of variable, the covariance matrix that will be used in the data assimilation algorithm is therefore $D_{A}$, it is diagonal. At the same time, the covariance information still has some interesting features, if the change of variable A is carefully chosen.

**Figure 1:**
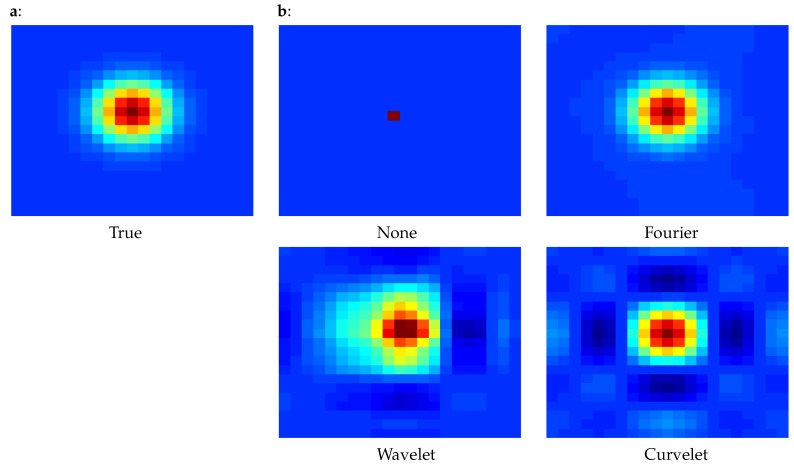
Correlation of the central observation point with respect to its neighbors. Dark red indicates values close to 1, blue is 0. (**a**) true correlation that we are trying to reproduce. (**b**) correlations obtained with the combination of a diagonal matrix and four different changes of variable: none (**top left**), change into Fourier space (**top right**), change into wavelet space (**bottom left**), change into curvelet space (**bottom right**).

As an illustration, Figure 1 presents the correlations of the central point with respect to its neighbors for diagonal covariance matrices using various changes of variables: none, change into wavelet space, change into Fourier space, change into curvelet space. This figure was produced using a diagonal correlation matrix D, then applying the chosen change of variable to obtain $R=ADA^{T}$, then plotting the correlation described by R. We can see in the figure that interesting correlations can be produced with an adequate change of variable. Indeed, all these changes of variables have the following fact in common: they perform a change of basis such that the new basis vectors have supports distributed over multiple neighboring points (contrary to the classical Euclidean basis vector, which are zero except in one point). This fact explains the fact that R is now non-diagonal.

Let us explain briefly the Fourier, wavelet and curvelet change of variables. For Fourier, the image is decomposed in the Fourier basis:

$$y=\sum_{j}\langle y,\varphi_{j}\rangle \varphi_{j}$$

where $\left(\varphi_{j}\right)$ represents the Fourier basis (e.g., sinusoidal functions) and the index *j* describes the scale of the *j*th basis vector (think of *j* as a frequency). The change of variables consists of describing y by its coefficients $y_{j}$ on the basis $\left(\varphi_{j}\right)$: $y_{j}=\langle y,\varphi_{j}\rangle$.

Similarly for the wavelets, the decomposition writes

$$y=\sum_{j,k}\langle y,\varphi_{j,k}\rangle \varphi_{j,k}$$

where $\left(\varphi_{j,k}\right)$ represents the wavelet basis (e.g., Haar or Daubechies), where the index *j* describes the scale of the *j*th basis vector and *k* is its position in space (think of wavelets as localised Fourier functions). The change of variables (A, denoted W for the wavelets) into wavelets space consists of describing y by its coefficients $y_{j,k}$ on the basis $\left(\varphi_{j,k}\right)$: $y_{j,k}=\langle y,\varphi_{j,k}\rangle$. In other words, Wy is the vector of coefficients ${\left(y_{j,k}\right)}_{j,k}$.

This is also similar for the curvelets:

$$y=\sum_{j,k,l}\langle y,\varphi_{j,k,l}\rangle \varphi_{j,k,l}$$

where the index *l* describe the orientation of the basis vector.

Using these changes of variables then allows various observation error modelling:

- Fourier: when the errors change with the scale only
- Wavelets: when the errors change with the scale as well as the position (e.g., for a geostationary satellite whose incidence angle impacts the errors, so that the errors vary depending on the position in the picture)
- Curvelets: when the errors change with the scale, the position and the orientation (e.g., when errors are highly non linear and depend on the flow, so that they are more correlated in one direction than another).

In this work, our focus is with wavelet basis, which presents many advantages: there exists fast wavelet transform algorithms (as for Fourier), so the computational cost remains reasonable. Also, contrary to Fourier, wavelets are localised in space and allow error correlations that are inhomogeneous in space, which is more realistic for satellite data, as well as data with missing zones.

To be more specific about wavelet transform, let’s assume the observation space is represented by a subset of Z, where each number represents a given observation point location (in 1D). Wavelet decomposition consists of computing, at each given scale, a coarse approximation at that scale, and finer details. Both are decomposed on a multiscale basis and are therefore represented by their coefficients on the bases. Approximation and details coefficients are given by a convolution formula:

$$c^{j-1}\left[n\right]=\sum_{p\in Z}h\left[p-2n\right]c^{j}\left[p\right];\;d^{j-1}\left[n\right]=\sum_{p\in Z}g\left[p-2n\right]d^{j}\left[p\right]$$

where $c^{j}\left[n\right]$ represents the approximation coefficient at scale *j* at point n∈Z, $d^{j}\left[n\right]$ represents the details coefficient at scale *j* at point n∈Z, *h* and *g* are functions depending on the chosen wavelets basis, each of them being equal to zero outside of their support $\left[n_{1};n_{2}\right]$. Moreover, wavelet *g* has *k* vanishing moments. A wavelet is said to have a vanishing moment of order k if *g* is orthogonal to every polynomial of degree up to k−1. As an example a wavelet with 1 vanishing moment is represented by a filter *g* such that $\sum_{n}g\left[n\right]=0$. This property is very important. Indeed, if the correlation is smooth enough (i-e. can be well approximated by polynomials of degree smaller than *k*), then details coefficients have a very small variance.

**Figure 2:**
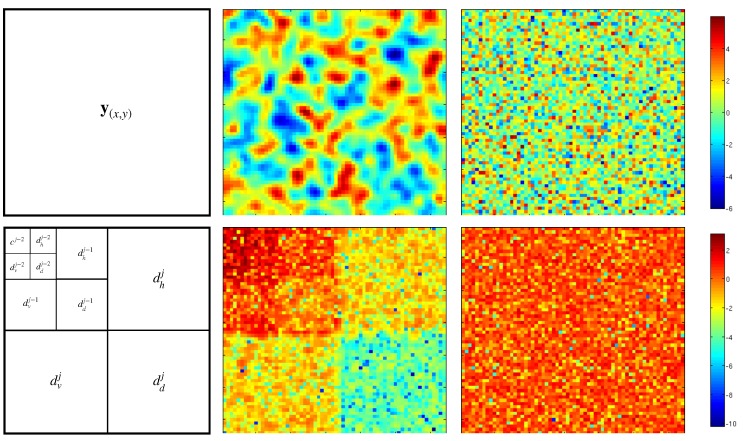
Top middle (resp right) panel shows an example of correlated (resp uncorrelated) noise. On bottom left, the scheme of the organisation of wavelet coefficient with three scales. On bottom middle (resp right), the logarithm of the absolute value in wavelet space of the correlated (resp uncorrelated) noise. We can see that approximation coefficient ($c^{j-2}$) are significantly larger than small scale details coefficients ($d_{*}^{j}$).

This can be extended in 2D (or more), where details coefficients at scale *j* will be separated into 3 components: vertical ($d_{v}^{j}$), horizontal ($d_{h}^{j}$) and diagonal ($d_{d}^{j}$). Bottom-left panel of Figure 2 shows the classical representation of coefficients on the wavelet space of a 2D signal. Finer details coefficient being stored in the three larger submatrices. The coarse approximation at finer scale is stored in the top-left submatrix and is itself decomposed into details and a coarser approximation. In this example the signal is decomposed into three scales.

**Figure 3:**
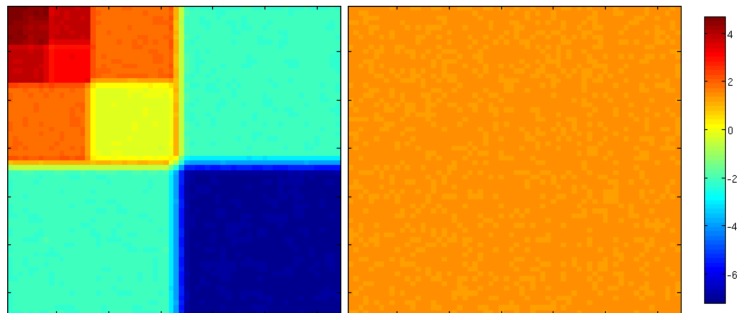
Logarithm of the variances of a correlated (**left**) and an uncorrelated (**right**) noise in the wavelet space. One can see that approximation coefficients (for a correlated noise) have a very small variance while approximation coefficients have a large variance. When no correlation exists in the noise, all the coefficients have the same variance (which is one in this example).

The top row of Figure 2 shows examples of both correlated (middle panel) and uncorrelated (right panel) noise. The bottom row shows their respective coefficient (in log-scale) in wavelet space using the representation depicted above. While uncorrelated noise affect all scales indiscriminately, the effect of correlated noise is significantly different from one scale to another (up to a factor 100 in this example). One can observe that approximation coefficients are very large compare to small scale details coefficients. This means that correlated noise (or smooth noise) affect more approximation coefficients than small scale details coefficients. This is due to the “vanishing moment” property of the wavelet *g*. Additionnaly the effect of a correlated noise resemble a (different) uncorrelated noise on each scale, meaning the diagonal approximation of the error covariance matrix will be a good one, as long as the sub-diagonals corresponding to each scales are different. This is represented in Figure 3 that shows the variances (log-scale) in the wavelet space of both correlated and uncorrelated noise from Figure 2.

In the next paragraphs we will describe hos this transformation can be used in the two classical frameworks of Data Assimilation: variational methods and filtering methods.

### 2.3. Implementation in Variational Assimilation

In the framework of variational assimilation, the analysis is set to be the minimizer of the following cost function *J*, which diagnoses the misfit between the observations and a priori information and their model equivalent, as in (2):

$$J\left(x_{0}\right)=J^{b}\left(x_{0}\right)+J^{o}\left(x_{0}\right),\;\;\mathrm{where}\;\;J^{b}=∥x_{0}-x_{0}^{b}{∥}_{B}^{2},\;J^{o}={∥H\left(x\left(x_{0}\right)\right)-y∥}_{R}^{2}$$

where $x(x_{0})$ is the solution of Equation (1), when the initial state is $x_{0}$. In practice y stores time-distributed observations, so that it can be written as

$$J^{o}\left(x_{0}\right)=\sum_{i\;\mathrm{obs}.\;\mathrm{time}\;}{∥H_{i}\left(x\left(x_{0}\right)\right)-y_{i}∥}_{R_{i}}^{2}$$

where $H_{i}$ is the observation operator at time *i*, $y_{i}$ is the observation vector at this time, and $R_{i}$ is the observation error covariance matrix.

Using the wavelets change of variables A=W, we choose a diagonal matrix $D_{w}$ (possibly varying with the observation time *i*, but we omit the index for the sake of simplicity), and we set

(3) $$J^{o}\left(x_{0}\right)=\sum_{i\;\mathrm{obs}.\;\mathrm{time}\;}{∥WH_{i}\left(x\left(x_{0}\right)\right)-Wy_{i}∥}_{D_{w}}^{2}$$

so that the observation error covariance matrix that is actually defined is:

(4) $$R^{-1}=W^{T}D_{w}^{-1}W$$

Meanwhile, the algorithm steps are:

1. Compute the model trajectory $x(x_{0})$ and deduce the misfits to observation $H_{i}\left(x\left(x_{0}\right)\right)-y_{i}$ for all *i*
2. Apply the change of variable (wavelet decomposition) $WH_{i}\left(x\left(x_{0}\right)\right)-Wy_{i}$
3. Compute the contribution to the gradient for all i: $\nabla J^{o}=H^{T}W^{T}D_{w}^{-1}\left(WH_{i}\left(x\left(x_{0}\right)\right)-Wy_{i}\right)$
4. Descent and update following the minimization process

In this algorithm, we can see that there is no need to form nor inverse R, the optimization module only sees the diagonal covariance matrix $D_{w}$, so that the minimization can be approached with classical methods like conjugate gradient or quasi Newton. Therefore, the only modification consists of coding the wavelets change of variable and its adjoint. As wavelet transforms are usually implemented using optimized and efficient libraries, the added cost is reasonable [2].

### 2.4. Implementation in Kalman Filtering

In this Section we explain the practical implementation of accounting for correlated observation errors in the Kalman filtering framework. We will briefly recall the main equations of the filters and then explain the adequate alterations to include observation error covariance modelling. We will use the standard notations and algorithms of data assimilation [8,15].

#### 2.4.1. Extended Kalman Filter

Using standard notation, the analysis step of the extended Kalman filter writes as follows:

(5) $$\begin{array}{ccc} x^{a} & = & x^{f}+K\left(y^{o}-H\left(x^{f}\right)\right)\\ K & = & P^{f}H^{T}{\left(HP^{f}H^{T}+R\right)}^{-1} \end{array}$$

where $x^{a}$ and $x^{f}$ are the analysis and forecast state vectors respectively, $y^{o}$ is the observation vector, H is the (possibly nonlinear) observation operator, H is its tangent linearized version, K is the Kalman gain matrix, $P^{f}$ is the forecast error covariance matrix and R is the observation error covariance matrix.

To account for a non diagonal R while keeping the algorithm easy to implement, let us assume that we define R as previously by (4), with $D_{w}$ a diagonal matrix whose dimension is *d*, the number of wavelet coefficients (equal to the observation space dimension *p*). We recall that the wavelet transform W is orthonormal, we have

$$W^{T}W=I_{d},\;W^{-1}=W^{T}$$

where $I_{d}$ is the identity matrix in dimension d=p. Then we can write the Kalman gain matrix as:

$$K=P^{f}H^{T}\;W^{T}{\left(WHP^{f}H^{T}W^{T}+D_{w}\right)}^{-1}W$$

In this equation, we can see that the algorithm complexity is preserved, up to a change of variable:

- the required matrix inversion ${\left(WHP^{f}H^{T}W^{T}+D_{w}\right)}^{-1}$ can be expected to be of the same complexity as ${\left(HP^{f}H^{T}+R\right)}^{-1}$,
- two changes of variables, and their inverse, are required, one on the matrix $HP^{f}H^{T}$, and one in the end for the matrix ${\left(WHP^{f}H^{T}W^{T}+D_{w}\right)}^{-1}$ to get the final Kalman gain.

As wavelet transforms are usually implemented using optimized and efficient libraries, we can also expect the added cost to be affordable. In particular efficient parallel libraries exist [16] and it is well suited for GPU computing.

#### 2.4.2. Stochastic Ensemble Kalman Filter

Let us recall the main ingredients of analysis step of the stochastic ensemble Kalman filter, as can be found in [8] (pp. 158–160). The number *m* stands for the number of members in the ensemble.

- A set of *m* perturbed observations is generated to account for the observation error:
  $$\mathrm{for}\;i=1..m,\;\mathrm{compute}\;\;y_{i}^{o}=y^{o}+u_{i},\;\;\mathrm{with}\;u_{i}∼N\left(0,R\right)$$
- The $Y_{f}$ matrix is computed. First we compute
  $$\bar{u}=\frac{1}{m}\sum_{i=1}^{m}u_{i}$$
  then the *i*th column of $Y_{f}$ is given by:
  $${\left[Y_{f}\right]}_{i}=\frac{H\left(x_{i}^{f}\right)-u_{i}-\left(H\left(\bar{x^{f}}\right)-\bar{u}\right)}{\sqrt{m-1}},\;\mathrm{for}\;i=1..m$$
- The Kalman gain matrix is computed:
  $$K=X^{f}Y_{f}^{T}{\left(Y_{f}Y_{f}^{T}\right)}^{-1}$$
- The analysis members are computed:
  $$x_{i}^{a}=x_{i}^{f}+K\left[y_{i}^{o}-H\left(x_{i}^{f}\right)\right],\;\mathrm{for}\;i=1..m$$

This algorithm can accommodated the accounting for observation errors correlations using this simple modification of the very first step (perturbation of the observations), which is the only occurrence of the R matrix:

$$\mathrm{for}\;i=1..m,\;\left\{\begin{array}{c} \beta_{i}∼N\left(0,I\right)\\ u_{i}=W^{T}D_{w}^{1/2}\beta_{i}\\ y_{i}^{o}=y^{o}+u_{i} \end{array}\right.$$

As we can see, this is quite easy to implement, as $D_{w}$ is diagonal, its square root is easily obtained. $W^{T}$ is the inverse of the wavelet transform. This operation has to be performed *m* times, possibly in parallel, so that the added cost should be negligible.

#### 2.4.3. Deterministic Ensemble Kalman Filter

As previously, let us recall the main ingredients of the deterministic Kalman filter (from [8] p. 162 and following), and see how it can be adapted to account for a change of variable in wavelet space for observation error covariance modelling. Let us first take a look at the analysis phase of the filter:

- Contrary to the stochastic Kalman filter, the $Y_{f}$ observation anomalies matrix is not perturbed:
  $${\left[Y_{f}\right]}_{i}=\frac{H\left(x_{i}^{f}\right)-\bar{y^{f}}}{\sqrt{m-1}}\;\;\mathrm{for}\;i=1..m,\;\;\mathrm{with}\;\;\bar{y^{f}}=\frac{1}{m}\sum_{i=1}^{m}H\left(x_{i}^{f}\right)$$
- The analysis is given by
  $$x^{a}=\bar{x^{f}}+K\left[y^{o}-H\left(\bar{x^{f}}\right)\right],\;\mathrm{for}\;i=1..m$$
- The Kalman gain matrix writes
  $$K=P^{f}H^{T}{\left(HP^{f}H^{T}+R\right)}^{-1}$$
- The analysis anomalies are given by
  $$w^{a}=Y_{f}^{T}{\left(Y_{f}Y_{f}^{T}+R\right)}^{-1}\delta ,\;\;\mathrm{with}\;\delta =y^{o}-H\left(\bar{x^{f}}\right)$$
  which can be rewritten using an adapted version of the Sherman-Morrison-Woodbury formula:
  $$w^{a}={\left(I_{m}+Y_{f}^{T}R^{-1}Y_{f}\right)}^{-1}\;Y_{f}^{T}R^{-1}\delta =T\;Y_{f}^{T}R^{-1}\delta ,\;\mathrm{with}\;T={\left(I_{m}+Y_{f}^{T}R^{-1}Y_{f}\right)}^{-1}$$
- And finally, here is the generation of the posterior ensemble:
  $$x_{i}^{a}=\bar{x^{f}}+X^{f}\left(w^{a}+\sqrt{m-1}{\left[T^{\frac{1}{2}}U\right]}_{i}\right)$$
  where $X^{f}$ is a matrix whose columns are the normalized forecast anomalies and U is an arbitrary orthogonal matrix:
  $${\left[X^{f}\right]}_{i}=\frac{x_{i}^{f}-\bar{x^{f}}}{\sqrt{m-1}},\;\mathrm{for}\;i=1..m$$

The required modifications to include the change of variable W are twofold:

1. First, we change variable in the expression $Y_{f}^{T}R^{-1}Y_{f}$:
  $$Y_{f}^{T}R^{-1}Y_{f}=Y_{f}^{T}W^{T}D_{w}^{-1}WY_{f}={\left(WY_{f}\right)}^{T}D_{w}^{-1}\left(WY_{f}\right)$$
  Here we can see that the matrix inversion still occurs with a diagonal matrix, as before, and we just have to apply the wavelet transform on $Y_{f}$, which is a matrix with *m* columns, where *m* is small.
2. Second, we change variable in the expression $Y_{f}^{T}R^{-1}\delta$:
  $$Y_{f}^{T}R^{-1}\delta ={\left(WY_{f}\right)}^{T}D_{w}^{-1}\left(W\delta \right)$$
  Here the change of variable is done only once on δ the inovation vector. It has been done previously for $Y_{f}$.

Notice that the matrix inversion $T={\left(I_{m}+Y_{f}^{T}R^{-1}Y_{f}\right)}^{-1}$, as well as computing its square-root $T^{\frac{1}{2}}$, takes place in ensemble subspace, of dimension *m*, and is therefore efficient even if the change of variable impacted $Y_{f}^{T}R^{-1}Y_{f}$.

### 2.5. Toward Realistic Applications

This approach works well with idealistic academic test cases. To go toward realistic applications, several issues need to be sorted out. In this section we address two of them. The first one is quite general and requires the ability to deal with incomplete observations, where part of the signal is missing, either due to sensor failure or external perturbation/obstruction. The second one is more specific to variational data assimilation, where the conditioning of the minimisation, and hence its efficiency, can be severely affected by complex correlation structure in the observation error covariance matrix. It is likely to also affect the Kalman Filter, in particular the matrix inversion in the observation space it requires (e.g., in Equation (5)), but it is yet to be demonstrated.

#### 2.5.1. Accounting for Missing Observations

When dealing with remote sensing, reasons for missing observation are numerous, ranging from a glitch in the acquisition process to an obstacle blocking temporarily one part of the view. This may be quite detrimental to our proposed approach since it violates the multi-scale decomposition hypotheses. However, contrary to Fourier, wavelets (and many, if not all, x-lets) have local support that may be exploited to handle this issue. Please note that the same kind of issue can arise in case of complex geometry. For instance if one observes sea surface temperatures, land is present in the observation, while not being part of the signal. Somehow it can be treated as missing value.

One possibility would be to use inpainting techniques to fill in the missing values. However, this would make the associated error very difficult to describe. Indeed, it would require the estimation of the errors associated with introducing ’new’ data in the missing zones, which is likely to be of different nature than that of original observations.

The idea is therefore to adapt the R matrix to the available data. Without any change of variable, the adaptation would be straightforward, as we would just have to apply a projection operator π to both the data and the R matrix:

$$y^{o}-H\left(x^{f}\right)\to \pi \left(y_{\pi }^{o}-H\left(x^{f}\right)\right);\;R\to \pi R\pi^{T}$$

where the projector π maps the full observation space into the subset of the observed points, and $y_{\pi }^{o}$ represents the full observation vector (with 0 where there is no available data).

**Figure 4:**
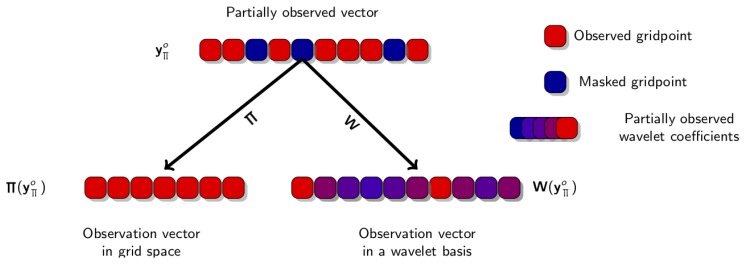
Schematic representation of the impact of non-observed pixels on the observation vector for both “pixel-grid” space and wavelet space. In pixel-grid space, missing pixels simply disappear. In wavelet space, missing pixels lead to partially observed wavelets coefficients.

When using a change of variable into wavelet space, it is a bit more tricky to perform, as a given observation point is used to compute many wavelet coefficients. Vice-versa a given wavelet coefficient is based on several image observation points. As a consequence, if some observation points are missing and others are available, it may result in “partially observed” wavelet coefficients, as schematized in Figure 4.

Our choice is to still take into account these coefficients (and not discard them, because it would result in discarding too much information, as a single missing observation point affects numerous wavelet coefficients), but to carefully tune the diagonal coefficient of the diagonal matrix $D_{w}$.

Missing observation points have two opposite effects:

- A missing observation point does not have signal nor any error, so we could expect the error variance of the impacted wavelet coefficients to decrease.
- A missing observation point leads to more discontinuities in the error signal as the error is 0 where a observation point is missing. This will increase significantly the fine scale coefficients, which is quite unfortunate since a good property of the wavelet decomposition was to have very small coefficient on finer scale (see end of Section 2.2).

To account for both effects, we propose an heuristic to adjust the variance $\sigma_{\pi }^{2}$ (in other words, the coefficients of the diagonal matrix $D_{w}$) corresponding to coefficients whose support is partially masked as follows:

$$\sigma_{\pi }^{2}=\left(\left(1-\beta \right)\sigma^{2}+\beta \sigma_{a}^{2}\right)\;I^{2}$$

where:

- $\sigma^{2}$ is the original error variance (e.g., given by the data provider);
- β∈[0,1] is multiplied by the variance of the wavelet coefficient without any correlation $\sigma_{a}$: it accounts to inflating the variance due to missing information (loss of the error averaging effect);
- I∈[0,1] stands for information content, and models the deflation effect. It takes into account the impact of missing observation points on the considered wavelet coefficient.

We now explain how β and *I* can be tuned. For the sake of simplicity, let us assume that our observation lives in a one dimensional space.

##### Computation of *I*

The deflation percentage *I* for each coefficient is computed using also a wavelet transform, where *h* and *g* are replaced by constant functions with the same support:

$$h^{0}\left[n\right]=\frac{1}{n_{2}-n_{1}+1};\;g^{0}\left[n\right]=\frac{1}{n_{2}-n_{1}+1};\;\forall n\in \left[n_{1};n_{2}\right]$$

Those functions extract the percentage of observation point present on the wavelet support. We proceed as follows. First we set the mask corresponding to the missing observation, it is an observation vector *m* equals to 1 where the observation point is observed and equal to zero where the observation point is missing. The wavelet transform of the mask aims to keep track of the impact of any missing observation point on any given wavelet coefficient. The percentage *I* is computed for each coefficient $c^{j}\left[n\right]$ and $d^{j}\left[n\right]$ by induction:

- At the finest scale $j_{max}$:
  $$\left\{\begin{array}{ccc} I(c^{j_{max}}\left[n\right]) & = & \sum_{p\in Z}h^{0}\left[p-2n\right]m\left[p\right]\\ I(d^{j_{max}}\left[n\right]) & = & \sum_{p\in Z}g^{0}\left[p-2n\right]m\left[p\right] \end{array}\right.$$
- At the other scales:
  $$\left\{\begin{array}{ccc} I(c^{j-1}\left[n\right]) & = & \sum_{p\in Z}h^{0}\left[p-2n\right]I\left(c^{j}\left[p\right]\right)\\ I(d^{j-1}\left[n\right]) & = & \sum_{p\in Z}g^{0}\left[p-2n\right]I\left(c^{j}\left[p\right]\right) \end{array}\right.$$

##### Computation of β

Let us now explain how to compute the inflation coefficient β. As explained previously, as *g* has *k* vanishing moments, small scale coefficients have small variances. However, when using masked signal, one loses this property. In other world, missing data damages the smoothness of the signal (and of the noise), which in turn damages the efficiency of wavelet representation. The coefficient β reflect the loss of the first vanishing moment: in the following formula we can see that β is zero if the first vanishing moment is preserved, and non-zero if not, in order to inflate the variance of small scales. For the finest scale, β is given by:

$$\left\{\begin{array}{ccc} c_{m}^{j_{max}}\left[n\right] & = & \sum_{p\in Z}\frac{\left|h[p-2n]\right|}{\sum_{q\in Z}\left|h\left[q-2n\right]\right|}m\left[p\right]\\ \beta_{m}^{j_{max}}\left[n\right] & = & \left|\sum_{p\in Z}\frac{g[p-2n]\;m[p]}{\sum_{q\in Z}\left|g\left[q-2n\right]\right|\;m\left[q\right]}\right| \end{array}\right.$$

Indeed, $\sum_{p\in Z}g\left[p-2n\right]m\left[p\right]=0$ means that wavelet still has a 0-th order null moment, even with missing coefficients, and in that case β=0.

Coarser scales coefficients are computed by induction, as:

$$\left\{\begin{array}{ccc} c_{m}^{j-1}\left[n\right] & = & \sum_{p\in Z}\frac{\left|h[p-2n]\right|}{\sum_{q\in Z}\left|h\left[q-2n\right]\right|}c_{m}^{j}\left[p\right]\\ \beta_{m}^{j-1}\left[n\right] & = & \left|\sum_{p\in Z}\frac{g[p-2n]\;m[p]}{\sum_{q\in Z}\left|g\left[q-2n\right]\right|\;m\left[q\right]}c_{m}^{j}\left[p\right]\right| \end{array}\right.$$

Finally, the variance model is modified as follows for every detail coefficient whose data is partially missing:

$$\sigma_{\pi }^{2}\left(d^{j}\left[n\right]\right)=\left(\sigma^{2}+\beta_{m}^{j}\left[n\right]\sigma_{a}^{2}\right)\;I{\left(d^{j}\left[n\right]\right)}^{2}$$

For approximation coefficient, only the deflation factor is used:

$$\sigma_{\pi }^{2}\left(c^{j}\left[n\right]\right)=\sigma^{2}\;I{\left(d^{j}\left[n\right]\right)}^{2}$$

Indeed, when the error is correlated, the variance of the approximation coefficient $\sigma^{2}$ is much greater than $\sigma_{a}^{2}$. This is the case on Figure 3 where $\sigma^{2}∼100$ while $\sigma_{a}^{2}=1$. Moreover, *h* can be seen as a local smoothing operator ($\sum_{n}h\left[n\right]=1$) and therefore correlated errors do not compensate themselves. Consequently, their is no need for inflation. Inversely, for finer details, in our example $\sigma^{2}∼10^{-2},10^{-4}$ so β has a significant impact on those scales.

These modifications give therefore a new diagonal matrix $D_{w}$ which takes into account the occurrence of missing information. Section 3 will present numerical results.

#### 2.5.2. Gradual Assimilation of the Smallest Scales

As will be shown in the numerical results Section 3 below, another issue can occur with real data: convergence issues due to the nature of observation errors. Indeed, what our experiments highlight is that our test-case behaves well when the represented error correlation are Gaussian and homogeneous in space. For correlated Gaussian errors whose correlations are inhomogeneous in space, convergence issues occur to the point that it destroys the advantage of using wavelets: they do worse than the classical diagonal matrix without correlation. Please note that in a general case, even accounting for homogeneous noise may degrade the conditioning of the minimization [4]. Wavelet transform does not change the conditioning of the problem, but its multi-scale nature can be of help to circumvent this problem.

Numerical investigation of the results shows that some sort of aliasing occurs for small wavelet scales. Indeed, smallest scales are the least affected by the correlated noise, so they are not well constrained by the assimilation and they tend to cause a divergence when large scales are not well known either, which is at the beginning of the assimilation iteration process. Removing the smaller scales altogether is not a suitable solution, as they contain valuable information we still want to use. The proposed solution is therefore to first assimilate the data without the small scales and then add smaller scales gradually. Please note that this is not a scale selection method per se, as all scales will eventually be included. It can be related to the quasi-static approach [17] that gradually include observations over time.

##### Description of the Gradual Scale Assimilation Method

Let us rewrite the observation cost function given by Equation (3):

$$\begin{array}{cc} J^{o}\left(x_{0}\right) & =\sum_{i\;\mathrm{obs}.\;\mathrm{time}\;}{∥WH_{i}\left(x\left(x_{0}\right)\right)-Wy_{i}∥}_{D_{w}}^{2}\\ & =\sum_{i\;\mathrm{obs}.\;\mathrm{time}\;}\sum_{s\;\mathrm{scale}\;}\sum_{k}\frac{|d_{y_{i}}^{s}\left[k\right]-d_{H_{i}\left(x\right)}^{s}{\left[k\right]|}^{2}}{\sigma_{s,k}^{2}} \end{array}$$

where $d_{y_{i}}^{s}\left[k\right]$, for k∈Z, (resp. $d_{H_{i}\left(x\right)}^{s}\left[k\right]$) represent the wavelet coefficients at scale *s* of the signal $y_{i}$ (resp. $H_{i}\left(x\right)$) and the $\sigma_{s,k}^{2}$ are the associated error variances (corresponding to the diagonal coefficients of the matrix $D_{w}$).

Let us denote by $J_{s,i}^{o}$ the total cost corresponding to the scale *s* and observation time *i*:

$$J_{s,i}^{o}=\sum_{k}\frac{|d_{y_{i}}^{s}\left[k\right]-d_{H_{i}\left(x\right)}^{s}{\left[k\right]|}^{2}}{\sigma_{s,k}^{2}}$$

We then decide that the information at a given scale is usable only if the cost remains small, e.g., smaller than a given threshold $\tau_{s}$, we define the thresholded cost $J_{s,i,\tau_{s}}^{o}$ by:

$$J_{s,i,\tau_{s}}^{o}=\left\{\begin{array}{cc} J_{s,i}^{o} & \mathrm{if}\;J_{s,i}^{o}\leq \tau_{s}\\ \tau_{s} & \mathrm{otherwise} \end{array}\right.$$

The new observation cost function is then:

$$J_{\tau_{s}}^{o}\left(x_{0}\right)=\sum_{i\;\mathrm{obs}.\;\mathrm{time}\;}\sum_{s\;\mathrm{scale}\;}J_{s,i,\tau_{s}}^{o}$$

As mentioned before, the same issue could arise when using Kalman Filter type techniques during the matrix inversion needed when computing the gain matrix. Similar approaches based on iterative and multi-resolution could be used to sort this out.

### 2.6. Experimental Framework

Numerical experiments have been performed to study and illustrate the two issues that were previously highlighted: how to account for covariances with missing observations, and how to improve the algorithm convergence while still accounting for smaller scale information. This paragraph describes the numerical setup which has been used.

We wish to avoid adding difficulty to these already complex issues, therefore we chose a so-called *twin experiment* framework. In this approach, synthetic observations are created from a given state of the system (which we call the “true state”, which will serve as reference) and then used in assimilation.

The experimental model represents the drift of a vortex on the experimental turntable CORIOLIS (Grenoble, France), which simulates atmospheric vortices in the atmosphere: the turning of the table provides an experimental environment which emulates the effect of the Coriolis force on a thin layer of water. A complete rotation of the tank takes 60 seconds, which corresponds to one Earth rotation.

#### 2.6.1. Numerical Model

A numerical model represents the experiment, using the shallow-water equations on the water elevation h(x,y,t) and the horizontal velocity of the fluid w(x,y,t)=(u(x,y,t),v(x,y,t)), where *u* and *v* are the zonal and meridional components of the velocity. The time variable *t* is defined on an interval $\left[t_{0},t_{f}\right]$, while the space variable (x,y) lives in Ω a rectangle in the plane $R^{2}$. The equations write:

$$\left\{\begin{array}{ccc} \partial_{t}u-\left(f+\zeta \right)v+\partial_{x}B & = & -ru+\kappa \Delta u\\ \partial_{t}v+\left(f+\zeta \right)u+\partial_{y}B & = & -rv+\kappa \Delta v\\ \partial_{t}h+\partial_{x}\left(hu\right)+\partial_{y}\left(hv\right) & = & 0. \end{array}\right.$$

The relative vorticity is denoted by $\zeta =\partial_{x}v-\partial_{y}u$ and the Bernoulli potential by $B=gh+\frac{u^{2}+v^{2}}{2}$, where *g* is the gravity constant. The Coriolis parameter on the β-plane is given by $f=f_{0}+\beta y$, κ is the diffusion coefficient and *r* the bottom friction coefficient. The following numerical values were used for the experiments: $r=9.10^{-7}$, κ=0, $f_{0}=0.25$, g=9.81 and β=0.0406. The model is discretized using a finite differences scheme over a 128×128 grid and a 4^*th*^-order Runge-Kutta scheme in time, with a time step of 2.5s. Please note that this means the model fields can be decomposed in up to 7 different scales using wavelet transform ($128=2^{7}$).

Additional equations represent the evolution of the tracer concentration (fluorescein):

(6) $$\left\{\begin{array}{c} \partial_{t}q+\nabla q\cdot w-\nu_{T}\Delta q=0\\ q\left(t_{0}\right)=q_{0}. \end{array}\right.$$

where $q_{0}$ is the initial concentration of the tracer (assumed to be known), $\nu_{T}=10^{-5}$ is the tracer diffusion coefficient and w=(u,v) the fluid velocity computed above.

#### 2.6.2. Synthetic Observations for Twin Experiments

In the twin experiment framework, observations are computed using the model. A known “true state” is used to produce a sequence of images which constitutes the observations. Therefore, the observation operator H is given by:

(7) $$H\left(x_{i}\right)=q\left(t_{i}\right).$$

where $q(t_{i})$ comes from (6).

Then assimilation experiments are performed starting from another system state, using synthetic observations. The results of the analysis can then be compared to the synthetic truth.

**Figure 5:**
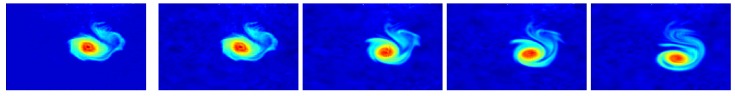
“True” initial concentration of the passive tracer (**1st left**) and noisy observations at initial time (**2nd**), after 90 min (**3rd**), 150 min (**4th**) and 270 min (**right**).

Unless otherwise stated, the assimilation period will be of 144 min, with one snapshot of passive tracer concentration every 6 min (24 snapshot in total). A selection of such snapshots is shown in Figure 5.

The observations are then obtained by adding an observation error $y=y^{t}+\epsilon$, with ϵ∼N(0,R) and R a suitably chosen matrix.

Our experiments will focus on three different formulations of the observation error covariance matrix. We will refer to “Pixels” the experiments for which there is no change of variable and the observation error covariance matrix is equal to D=diag(R). “Wavelet” will represent the experiments with the wavelet change of variable W and the observation error covariance matrix $D_{w}=\mathrm{diag}\left(WRW^{T}\right)$. Finally, the last set of experiments will proceed as for the wavelets but will adjust the observation error covariance matrix according to the computations presented in Section 2.5.1 and Section 2.5.2. The following Table 1 summarises this up.

## 3. Results

### 3.1. Accounting for Missing Observations

**Figure 6:**
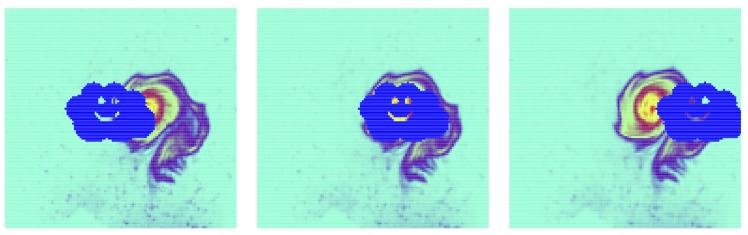
Example of an image sequence of the passive tracer concentration with missing observations. Left: first observation in the sequence, right: last observation.

Figure 6 provides an example of image data with 10% missing observations. It represents three images from an temporal observation sequence, in which we simulated the presence of a passing cloud. This sequence has been generated using the experimental model presented above, and the masking cloud is advected at a regular pace.

This image sequence was then modified by a strong additive and spatially correlated homogeneous and isotropic noise (signal to noise ratio SNR = 14.8 dB). Then we performed many twin data assimilation experiments, while varying two parameters:

- the covariance error matrix: diagonal in observation space, diagonal in wavelet space (no adjustment), diagonal in wavelet space and modified according to Section 2.5.1 (see Table 1);
- the percentage of occulted signal: varying from 0 to 18% (with varying cloud sizes). For each experiment, the passing cloud has the same shape but different sizes.

**Figure 7:**
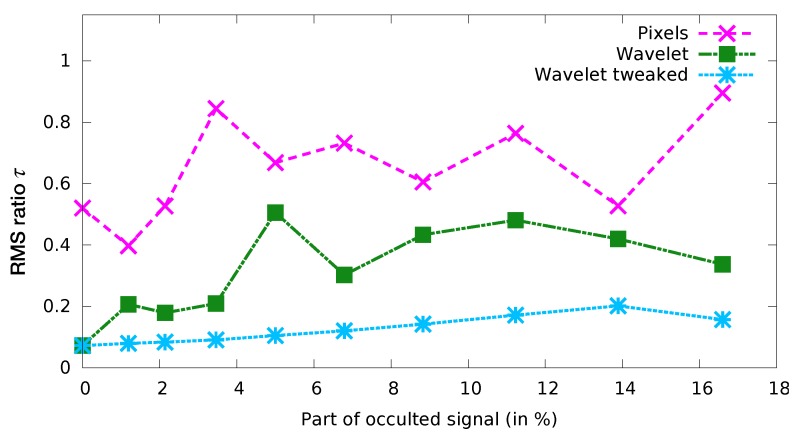
Ratio τ between the analysed state RMSE and the background RMSE for observations distorted by a strong spatially correlated additive noise, as a function of the percentage of missing observation points.

For each experiment, we computed τ the ratio between the root mean square error for the analysis and the background:

$$\tau =\frac{\mathrm{RMSE}(\mathrm{analysis})}{\mathrm{RMSE}(\mathrm{background})}=\frac{∥\left(h_{0}^{t},w_{0}^{t}\right)-\left(h_{0}^{a},w_{0}^{a}\right)∥}{∥\left(h_{0}^{t},w_{0}^{t}\right)-\left(h_{0}^{b},w_{0}^{b}\right)∥}$$

where $\left(h_{0}^{t},w_{0}^{t}\right)$, $\left(h_{0}^{a},w_{0}^{a}\right)$ and $\left(h_{0}^{b},w_{0}^{b}\right)$ represent the true, analysed and background initial states of the experimental system. This ratio is close to zero when the analysis is much closer to the true state than the background (which represents the “no assimilation” state), and close to 1 when the analysis performs poorly. Figure 7 shows the resulting ratios for all the experiments. We can draw the conclusion that modifying the covariance matrix as proposed allows a considerable improvement from other methods, as it keeps the error below 20%, even for a widely occulted image sequence, despite the high noise level.

**Figure 8:**
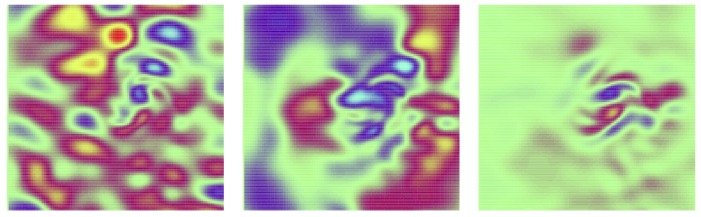
Error between the true velocity *v* and the analysed velocity after assimilation of an observation sequence with strong spatially correlated additive noise, with 9% missing observation points. The color scale ranges from −0.01 m.s${}^{-1}$ (**blue**) to 0.01 m.s${}^{-1}$ (**red**), which amounts to a third of the maximum velocity (ranging from −0.028 m.s${}^{-1}$ to 0.028 m.s${}^{-1}$). Left: result for the pixel method, middle: wavelet without modification, right: improved wavelet method.

Figure 8 gives more details for the experiments with 9% occulted signal, as it represents (as a function of the spatial variable $x\in \Omega \subset R^{2}$, see Section 2.6.1 for more details) the errors $v^{t}\left(x,0\right)-v^{a}\left(x,0\right)$, where $v^{t}\left(x,0\right)$ is the true longitudinal velocity at time 0 and $v^{a}\left(x,0\right)$ is the analysed longitudinal velocity at time 0. From this figure we can confirm that the modified wavelet covariance matrix does a much better job in approximating the true state.

### 3.2. Gradual Assimilation of the Smallest Scales

**Figure 9:**
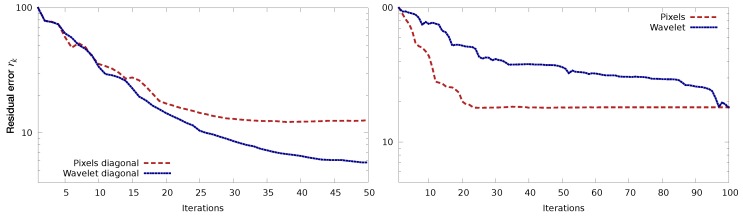
Ratio of the residual errors as a function of minimisation iterations for both wavelet and pixel methods, in the presence of correlated observation errors. (**Left**) homogeneously correlated error, (**right**) inhomogeneously correlated error.

Figure 9 illustrates the issue that we try to tackle using gradual assimilation. This figure presents the ratio $r_{k}$ of the residual errors, as a function of the iteration number *k*:

$$r_{k}=\frac{∥\left(h_{0}^{t},w_{0}^{t}\right)-\left(h_{0}^{k},w_{0}^{k}\right)∥}{∥\left(h_{0}^{t},w_{0}^{t}\right)-\left(h_{0}^{b},w_{0}^{b}\right)∥}$$

As before $\left(h_{0}^{t},w_{0}^{t}\right)$ and $\left(h_{0}^{b},w_{0}^{b}\right)$ represent the true and background initial states of the experimental system. Index *k* represents the iteration number (loop index in the assimilation process) and $\left(h_{0}^{k},w_{0}^{k}\right)$ is the initial state vector computed by the assimilation system after *k* iterations. Both panels of Figure 9 shows the evolution of these ratio as a function of *k* for the “Pixels” and the “Wavelet” methods for covariance matrices modelling, as described in Table 1. The difference lies in the actual error that is added to the observations:

- on the left panel, the error is as previously described: $y=y^{t}+\epsilon$, with ϵ∼N(0,R), it is spatially correlated but the correlation is homogeneous in space;
- on the right panel, we added an inhomogeneoulsy correlated error: $\epsilon =W^{T}D_{w}^{1/2}\beta$ with β∼N(0,I).

As we can see on the left panel, accounting for correlated observations thanks to the “Wavelet” method is beneficial for an homogeneously correlated noise, as the error is much decreased than for the “Pixels” method, for which no error correlation is modelled. However, when the error correlation is not homogeneous, the “Wavelet” method, despite with the correct error covariance matrix, fails to do better than the “Pixels” method.

**Figure 10:**
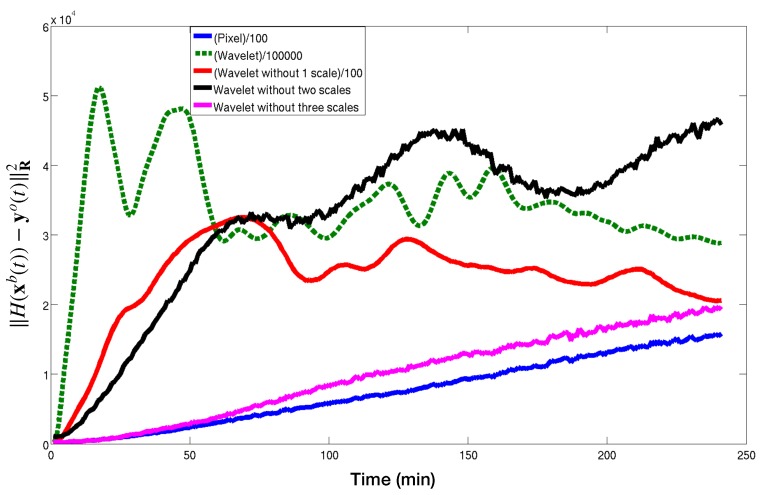
Discrepancy between the background (**no assimilation**) concentration trajectory and the successive observations along time. The discrepancy measurement is computed using the norms given by the observation term of the cost function for various methods: pixel (**solid blue**), classical wavelet (**dashed green**), wavelet excluding the finest scale (**solid red**), wavelet excluding the two finest scales (**solid black**), wavelet excluding the three finest scales (**solid pink**).

To investigate the issue, Figure 10 presents the discrepancy between the background and successive observations for various time:

$$∥y_{t_{i}}-H\left(x_{0}^{b}\right){∥}_{X}^{2},\;\;\mathrm{for}\;0\leq i\leq 240$$

with X=D=diag(R) for Pixels and $X=D_{w}=\mathrm{diag}\left(WRW^{T}\right)$ for Wavelet. It suggests an issue probably similar to what we could call aliasing of the smallest scales. Indeed let us examine more closely this figure.

On the one hand, the blue line represents this norm for the Pixel case. It starts with a small value (the only difference comes from the noise) and, as time goes by, the vortex drifts and the difference with the initial concentration steadily increases. As one would expect, the farther the vortex drift, the higher the difference with the initial concentration is, all the scales being given the same uncertainties.

On the other hand, the wavelet-based norm (in green), shows a steep increase at the beginning, but then oscillate around a ’plateau’. This happens because, at this point, the norm is really dominated by the small scales. Indeed, the smallest scales are the least affected by the correlated noise. Therefore their associated error variances are the smallest (i.e., one trusts more the small scales). As it is the inverse of the variances that is used as a weight in the norm, it should be expected that they dominate the norm. However it prevents to discriminate between two large scale signals, when the difference is too large (when the green curve stop being monotonic), so the minimisation problem becomes ill-posed.

Red, black and purple curves show the same quantity as the green one, but removing the 1, 2 and 3 finest scales in the multi scale decomposition respectively. The problem appears later (i.e., for larger discrepancies) when removing the finest scales and even disappear for the purple one. This motivates the introduction of the gradual assimilation of the smallest scales we presented above in Section 2.5.2.

**Figure 11:**
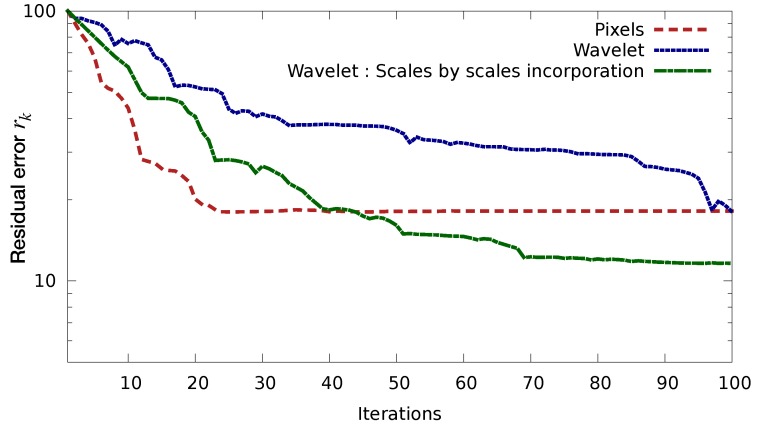
Ratio of the residual error as a function of minimisation iterations in the presence of inhomogeneously correlated observation errors, for three methods: pixels (**dashed red**), classical wavelet (**dotted blue**), modified wavelet (**dashdotted green**).

Figure 11 is similar to the right panel of Figure 9, where we added the “Wavelet scales by scales” method. The green curve shows the evolution of the residual error for this method, with $\tau_{s}=4.5$ for all *s*. This value has been chosen to preserve Gaussianity in the retained scales. Indeed, for a Gaussian signal 99% of the considered population should lie within 3 std dev of the mean (here it is a square and divided by two, hence 4.5). As we can see, this method clearly improves the above-mentioned issue, as the convergence is reasonably good and the error improved.

**Figure 12:**
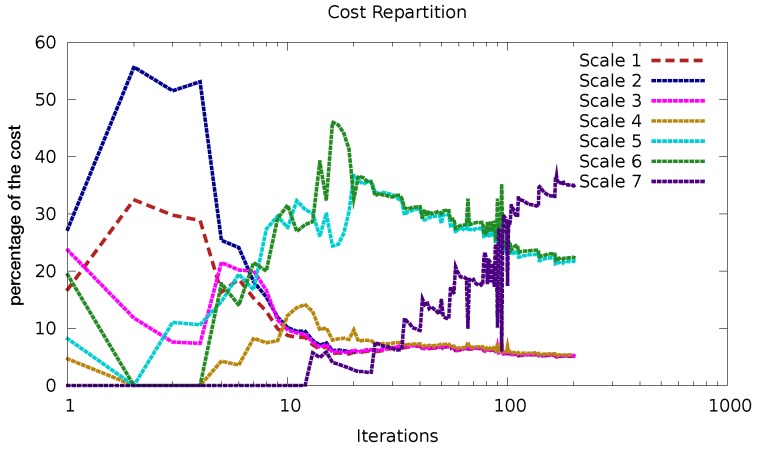
Contribution to the observation cost function, in percentage, of each activated scale, as a function of the minimisation iterations. The coarsest scale is the first one, the finest scale is the 7th.

Figure 12 gives more details about how the minimization actually operates. It shows the contribution to the observation term of the cost function from each activated scale. The coarser scales are dominating at the very beginning of the minimisation and converge quite quickly (after 10 iterations), then scales 5 and 6 dominate and converge after 100 iterations. The finer scale (scale 7) appears later and is gradually assimilated (image by image) and has not fully converged yet after 200 iterations.

## 4. Discussion

In this paper, we addressed an important yet often overlooked aspect of data assimilation: how to account for correlations in observation errors statistics. This question is a known obstacle of operational assimilation, as it implies technical as well as conceptual difficulties.

In this regard, we proposed an extension of the previous study [2], using wavelets transform in order to account for correlated observation errors in variational assimilation as well as Kalman filtering.

Keeping in mind the objective of using this methodology for real, operational, data assimilation, we choose to address two difficulties: accounting for missing observations (e.g., passing clouds for ocean color images) and scale-progressive assimilation in order to make the most of the multiscale aspect of the wavelet transform and improve convergence. For these two aspects we developed appropriate methodologies, which proved satisfactory to address both issues.

These promising results open new possibilities for accounting for correlated errors in operational data assimilation, e.g., regarding the following applications:

- Assimilation of the SWOT data (Surface Water and Ocean Topography): SWOT satellite (operational in 2021) has a large swath and will produce altimetric data for the ocean. Because of the swath width, any tiny oscillation of the satellite will have a wide impact on the observation error correlation that are therefore complex (inhomogeneous). The images are supposed to be filtered in order to avoid any problem. Our method could help to fully use the data without filtering out valuable information.
- Assimilation of ocean color images (imaging phytoplankton, in marine biology and ocean model coupling), for which the images are damaged by passing clouds.
- Any other application domain with dense observations, correlated errors, partially missing observations.

## Figures and Tables

**Table 1:** Summary of the experiments description: name, change of variable, observation error covariance matrix.

Experiment Name	Change of Variable	Observation Error Covariance Matrix
Pixels	none (identity)	D=diag(R)
Wavelet	W	$D_{w}=\mathrm{diag}\left(WRW^{T}\right)$
Wavelet tweaked	W	$D_{w}$ modified according to Section 2.5.1
Wavelet scale by scale	W	$D_{w}$ modified according to Section 2.5.2

## Abbreviations

The following abbreviations are used in this manuscript:

DA	Data Assimilation
SNR	Signal to Noise Ratio
RMSE	Root Mean Square Error
